# 3D Hippocampal Place Field Dynamics in Free-Flying Echolocating Bats

**DOI:** 10.3389/fncel.2018.00270

**Published:** 2018-08-23

**Authors:** Melville J. Wohlgemuth, Chao Yu, Cynthia F. Moss

**Affiliations:** Department of Psychological and Brain Sciences, Johns Hopkins University, Baltimore, MD, United States

**Keywords:** hippocampus, echolocation, neuroethology, active sensing, theta rhythm

## Abstract

A large body of laboratory research has investigated the process by which environmental cues are acquired and used for spatial navigation in rodents; however, the key to differentiating between species specializations and general principles lies in comparative research. Rodent research has focused on a class of neurons in the hippocampus implicated in the representation of *allocentric* space – termed place cells – and the process by which these representations form. One class of models of hippocampal place field formation depends on continuous theta, a low frequency brain oscillation that is prevalent in crawling rodents. Comparative studies of hippocampal activity in echolocating bats have reported many findings that parallel the rodent literature, but also describe noteworthy species differences, especially with respect to theta rhythm. Here, we first discuss studies of the bat hippocampal formation and point to gaps in our knowledge, which motivate our new lines of inquiry. We present data from the free-flying laryngeal echolocating big brown bat, which shows 3-D place cells without continuous theta, similar to reports from the lingual echolocating Egyptian fruit bat. We also report findings, which demonstrate that the animal’s control over echolocation call rate (sensory sampling) influences place field tuning. These results motivate future comparative research on hippocampal function in the context of natural sensory-guided behaviors.

## Introduction

A wide variety of survival behaviors depend on the brain’s collection, storage, and retrieval of memories (Allen and Fortin, 2013). This holds for single episodes, such as the recall of a past event, (Tulving, 2002), to more complex processes, like memory-guided navigation (McNaughton et al., 2006; Bird and Burgess, 2008). Our understanding of brain mechanisms supporting spatial memory comes from decades of research (Rolls, 1999; Suzuki and Clayton, 2000; Yartsev and Ulanovsky, 2013; Moser et al., 2015; Rueckemann and Buffalo, 2017), with a focus on the role of the hippocampus in navigation (Moser et al., 2017). The majority of work on the hippocampus has come from studies of spatial navigation in rodents (Paul et al., 2009; Eichenbaum and Cohen, 2014; Moser et al., 2017; Jeffery, 2018), with some studies in human and non-human primates (O’Mara et al., 1994; Rolls, 1999; Ekstrom et al., 2003; Hampton et al., 2004; Dimsdale-Zucker et al., 2018), birds (Clayton and Krebs, 1994; Hampton et al., 1995; Lucas et al., 2004) and more recently in two species of echolocating bats (Ulanovsky and Moss, 2007, 2011; Yartsev and Ulanovsky, 2013; Geva-Sagiv et al., 2015; Sarel et al., 2017). Here, we review research on bat hippocampal function, identify gaps in the literature and present new data that begin to address open questions in the field.

Comparative studies of hippocampal spatial representations have revealed striking similarities across species but also noteworthy differences, particularly with respect to the prevalence of continuous hippocampal theta rhythm. The theta oscillation is a frequency band (∼5–11 Hz range) of the local field potential (LFP), thought to be important in the dynamics of place field representations (Hasselmo et al., 2002). Models assert that this oscillation is important to the synchronization of activity in the hippocampus and across functionally related structures, as well as directly implicated in the formation of grid cells and place cells (O’Keefe and Recce, 1993; Bland and Oddie, 2001; Hasselmo, 2005; Hasselmo et al., 2007; Paul et al., 2009). In the rodent, theta oscillations occur continuously while the animal moves through the environment (Vanderwolf, 1969; Bland, 1986). In contrast, recordings from non-human primates (Robinson, 1980; Stewart and Fox, 1991) and humans show differences in theta, compared with rodents. Recordings in humans navigating a virtual navigation task reveal oscillations at slightly slower frequencies than the theta rhythm reported in rodents (Araújo et al., 2002; Ekstrom et al., 2005; Cornwell et al., 2008). These slower oscillations (<5 Hz) in humans show increases in power that are correlated with increases in movement in both virtual (Ekstrom et al., 2005) and real navigation (Arnolds et al., 1980), as well as performance in a spatial navigation task (Cornwell et al., 2008). One difference between rodents and humans appears in the continuity of theta, with studies in humans showing only intermittent bouts (Ekstrom et al., 2005; Watrous et al., 2013). These results motivate further research into the role of hippocampal theta in humans and other species beyond rodents. Similar to findings in primates, crawling big brown bats (*Eptesicus fuscus*) (Ulanovsky and Moss, 2007), and in flying Egyptian fruit bats (*Rousettus aegyptiacus*) (Yartsev et al., 2011), show only intermittent bouts of theta during spatial navigation and exploration. Because the theta rhythm falls in the range of the wingbeat of the Egyptian fruit bat at ∼6 Hz (Norberg and Norberg, 2012), measurements of this oscillation could be masked by movement artifact in studies of this free-flying mammal, calling for comparative studies in other bat species whose wingbeat rates fall outside of the theta band.

Much has been learned about spatial representation in the hippocampal formation of the Egyptian fruit bat. Recordings in the hippocampus of the free-flying Egyptian fruit bat provided the first classification of 3D hippocampal place cells (Yartsev and Ulanovsky, 2013). Follow-up studies on head direction cells in the presubiculum of the hippocampal formation similarly identified 3D tuning in space, and showed that this representation is in a toroidal coordinate frame (Finkelstein et al., 2015). More recent studies reported that the hippocampus remaps its allocentric place representation when the Egyptian fruit bats switch between using vision and echolocation for navigation (Geva-Sagiv et al., 2016). These studies show that sensory modality influences neural coding in the hippocampus, and motivate future studies designed to investigate the role of active sonar sampling on hippocampal place cell tuning in free-flying bats.

Studies of hippocampal representation in big brown bats demonstrated that place fields grow broader over a time period of 500 msec after sonar vocalizations (Ulanovsky and Moss, 2011). This was discovered by recording hippocampal activity in bats crawling in a small (68 cm × 73 cm) arena. The authors proposed that place fields became more diffuse after echo returns from the environment ceased, but the fact is, sonar echoes from the walls of the small arena would have ceased entirely within the first few milliseconds post-vocalization. Another interpretation of their findings is that hippocampal place fields are stable when sensory information is reliable, and then expand as stored sensory information fades, suggesting that sensory information can alter place field spatial tuning over short time scales. This interpretation is further supported by work demonstrating a correlation in the number of sensory cues and place field tuning (Battaglia et al., 2004). In this study, rodents were run on either a sensory cue-sparse, or sensory cue-rich track (e.g., visual, olfactory, auditory, and tactile cues); and researchers found that place fields were smaller on the cue-rich track. Taken together, these prior results in bats and rodents suggest that instantaneous sensory inputs may indeed affect the properties of hippocampal neurons, with increased sensory sampling leading to sharper representations of hippocampal place cells.

The dynamics of place field tuning reported by Ulanovsky and Moss (2011) lead to the following question: Does the bat’s production of calls at a high rate contribute to a sharpened representation of space? In other words, is place field tuning tightest when a bat samples its environment at a high rate? We hypothesize that there is a general relationship between sensory sampling and spatial representation in mammals. This hypothesis applies to all animals that probe the environment on different time scales, for example visual animals that move their eyes to foveate and attend to objects distributed across space, and rodents that whisk to investigate their proximal environment. Hippocampal recordings from a free-flying laryngeal echolocator that dynamically adjusts its sonar calls in response to objects in the environment can be used to test this hypothesis.

The echolocating big brown bat serves as a powerful model to perform comparative work on spatial representation, with a particular focus on hippocampal function in the context of natural sensory-guided behaviors. The big brown bat adapts its sonar vocal rate as it inspects objects and navigates in the environment (Griffin, 1958; Ulanovsky and Moss, 2008; Moss et al., 2011; Kothari et al., 2014). An increase in vocal rate leads to an increase in the rate of echoes returning to the bat’s ears, and we can therefore analyze changes in hippocampal activity with respect to changes in sensory sample rate. Moreover, LFP recordings of the hippocampal theta rhythm in big brown bats can provide additional comparative data on the prevalence of this LFP band in a freely flying animal whose wingbeat rate is about 12 Hz, and therefore less likely to mask theta through movement artifacts.

We assert that comparative studies of spatial representation in mammals are key to advancing the understanding of both general mechanisms and species specializations. Toward this end, we present preliminary data that begin to fill the gaps in our knowledge of spatial representation in freely echolocating bats, which can lay the foundation for future comparative studies of the hippocampal formation.

## Materials and Methods

### Animal Subjects

Three wild-caught big brown bats (*Eptesicus fuscus*) served as subjects in the following studies. The bats were collected in the state of Maryland under a permit issued by the Department of Natural Resources and were housed in animal vivaria at the University of Maryland – College Park, and Johns Hopkins University. All procedures were approved by the Institutional Animal Care and Use Committees at the University of Maryland and Johns Hopkins University, where this research was conducted.

### Behavioral Training

**FIGURE 1:**
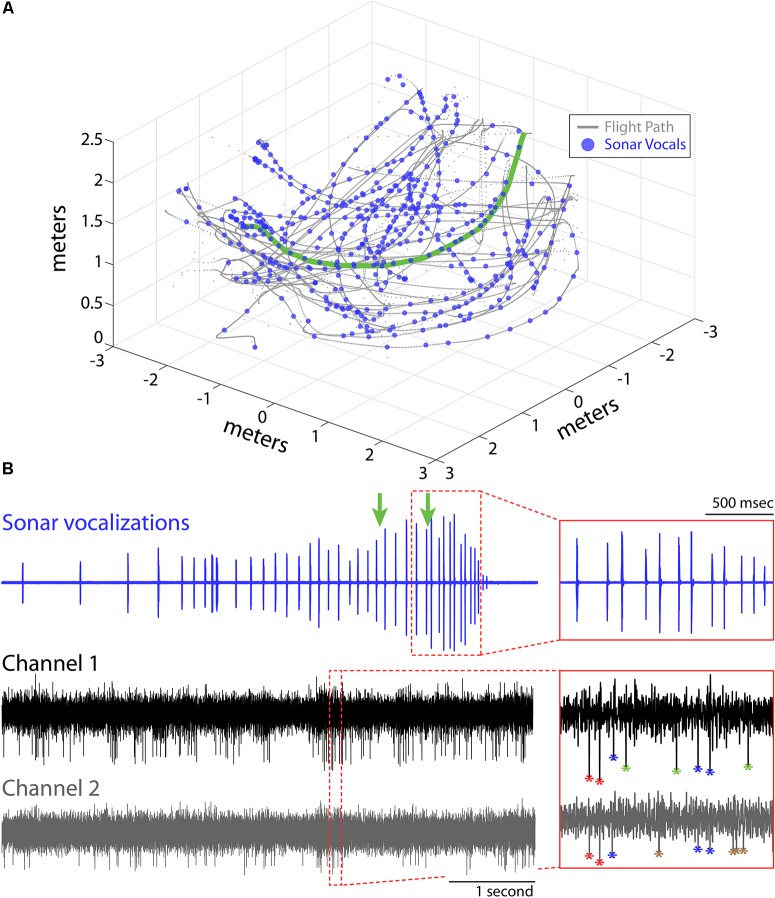
Experimental setup and recordings. **(A)** Flight trajectories (gray) and vocalizations (blue circles) during one experimental session for Bat 1. The bat navigated around obstacles (not shown) in a large flight room (6 m × 6 m × 3 m) while recordings of hippocampal activity, sonar vocalizations, and the animals’ flight paths were collected. **(B)** Example of sonar vocalizations (top), and two adjacent recording channels (middle and bottom) for one flight trial for the experimental session shown in **(A)** (selected flight trajectory shown in green in **A**). Green arrows mark the times when the bat passed the two flight obstacles in this trial. Insets show time expanded portions identified by the red, dotted boxes (top, vocals; bottom, neural). Action potentials from different neurons are color coded with different asterisks in bottom inset panel.

**FIGURE 2:**
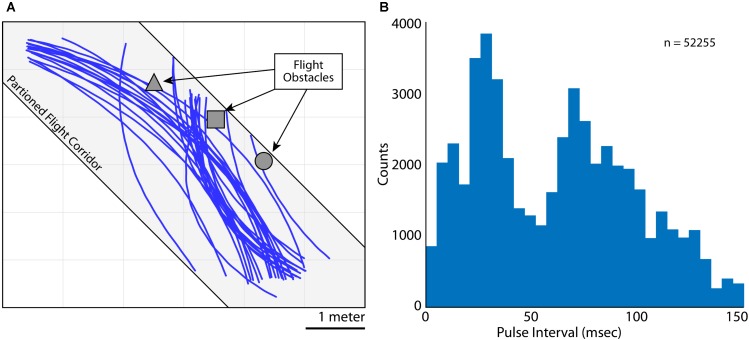
Flight and vocal behaviors. **(A)** 2D (X and Y) flight trajectories for Bat 3 during one experimental session. Flight paths are shown in blue, flight obstacles are shown in gray. The flight room was partitioned into a narrow corridor (gray shading, 6 m × 2 m × 3 m) to restrict the bat’s flight paths. **(B)** Distribution of pulse intervals (PIs) during all recording sessions for Bat 3. Note the bimodality of the distribution, indicating vocalizations emitted at a high rate (i.e., short PI’s) and lower rate (i.e., longer PI’s).

The three bats in this study performed similar flight tasks, involving free-flight behaviors within an experimental flight room, with and without flight obstacles. Bat 1 and Bat 2 were allowed unrestricted access to a large experimental flight room (**Figure 1A**, 6 m × 6 m × 3 m), while the flight paths of Bat 3 were restricted to a narrow corridor oriented along the diagonal of the flight room (**Figure 2A**, 6 m × 2 m × 3 m) to increase the coverage of space in which the bat flew. The corridor was constructed out of PVC mesh netting^1^ that created a flight barrier for the bat, but was acoustically transparent to the bats’ sonar vocalizations and resulting echoes. The PVC mesh was hung from the ceiling and secured to the floor so as to restrict the bats’ flight paths. In selected sessions (8/11 sessions for Bat 1, 6/11 sessions for Bat 2, and 8/16 sessions for Bat 3) obstacles were hung from the ceiling to provide objects for the bats to interact with while in flight. The flight obstacles for Bat 1 and Bat 2 were two cardboard boxes (28 cm × 20 cm × 18 cm). For Bat 3, we hung three different obstacles from the ceiling that were each made from dense foam: a square pyramid (15 cm × 15 cm × 30 cm), a cube (15 cm × 15 cm × 15cm), and a sphere (15 cm diameter). The flight obstacles served as proximal landmarks in the environment, while objects on the wall (e.g., cameras, computer, etc.) served as more distal landmarks throughout recording sessions. Recordings were made of the bats’ vocalizations using two Ultra Sound Advice microphones (sample rate = 250 kHz, band-pass filtered between 20 and 100 kHz) mounted above the floor. The bats’ flight trajectories were captured using a 16-camera, high-speed motion tracking system (Vicon), recording at 300 frames/sec, and calibrated to submillimeter accuracy by a moving 5-point wand at the beginning of each recording session. Three reflective markers were fixed to the bat (on top of the wireless neural transmitter), and these markers were tracked in 3D space to provide the location of the bat throughout the experiment. The same system was used to mark the locations of the flight obstacles, and ultrasonic microphones. Wireless neural recordings were collected in time synchrony with behavioral measurements using a neural telemetry device (Triangle Biosystems International, TBSI), and then digitized at 40 kHz (Plexon) and stored for offline analysis. An end trigger was used to provide a common time-stamp across hardware systems at the conclusion of each trial, when the bat landed. All experiments were performed in long wavelength illumination to preclude the bats’ use of vision for navigation (Hope and Bhatnagar, 1979).

### Electrophysiology

In order to record neural activity from a free-flying bat, we performed a surgery to implant an array of four tetrodes (16-channels, Neuronexus; 150 μm between shanks, 25 μm spacing between site centers) into the hippocampus. The tetrode array was mounted on a moveable microdrive, allowing us to advance and retract the array in order to locate the hippocampal cell layers. Rest period recordings were performed before and after experimental sessions to identify sharp waves as a functional indicator of our recording probe being in a hippocampal cell layer. Bat 1 flew for 11 sessions, Bat 2 for 11 sessions, and Bat 3 for 16 sessions. During each flight session, the bats flew for a minimum of 30 trials, and each trial was 10 s in length (Bat 1, average number of trials 34.3; Bat 2, average number of trials 35.6; and Bat 3, average number of trials 32.1).

### Data Analysis

#### Analysis of Sonar Vocal Behaviors

At the conclusion of the experiment, several analyses were performed in parallel on both the behavioral and hippocampal data. In order to determine the 3D location of the bats, data collected by our motion tracking hardware (Vicon/Nexus) was imported into Matlab, and separated into trials based on the end trigger generated during the experiment. We then analyzed the audio recordings of the bat’s vocalizations to find the onsets and offsets of each vocalization. This was done first by low-pass filtering the audio trace to calculate the amplitude envelope, and then using a threshold crossing set at 6 dB above the noise floor to identify each vocalization. The onsets were then corrected for the time of travel from the bat’s 3D position to the location of the microphone.

We calculated the pulse interval (PI) by measuring the time delay from the onset of one vocalization to the next. PI is used as a metric for vocal rate, with smaller PI’s indicating higher vocal rates. In order to determine if PI was significantly different across measured conditions (e.g., obstacles vs. no obstacles), we performed a permutation test. A permutation test was used because the distributions were not normal and failed the Lilliefors Test; we therefore chose a non-parametric test. For the permutation test, we collapsed all data across conditions into one group, and then randomly assigned data points (with replacement) into two groups that were equal in size to the original data sets. We then calculated the mean difference between the two randomly sorted groups. This was performed 1,000 times to calculate the distribution of randomized mean differences. If the actual mean difference between groups was greater than 95% of the calculated mean differences, the actual mean difference was considered significantly different.

#### Analysis of the Local Field Potential

Several different analyses were performed on the hippocampal data. To analyze the theta and delta bands of the LFP, we first low-pass filtered the wideband neural traces at 100 Hz. An FFT was then calculated to measure the power across frequencies in the 1–100 Hz range. In order to measure power in the delta and theta bands, the LFP was band-passed with an elliptical filter between 0.1–3 Hz and 5–11 Hz, respectively. Theta bouts were then defined, as in previous reports (Yartsev et al., 2011), when the power of the theta band was more than two times the power of the delta band in 1-s time bins. To calculate the proportion of time theta bouts occurred, we divided the total trial time by the time elapsed during theta bouts.

#### Construction of Place Maps

Wideband neural recordings were also filtered between 300 and 3,000 Hz to analyze spiking activity. We identified spiking activity through a mix of traditional tetrode clustering techniques (i.e., spike amplitude across channels) (Ulanovsky and Moss, 2007; Yartsev and Ulanovsky, 2013), as well as through single-channel wavelet clustering techniques (see **Figure 1B**, inset) (Quiroga et al., 2004). The wavelet clustering technique was used for spiking activity that was only found on one channel of the tetrode, and therefore precluded an analysis of spike amplitude across channels as a clustering method.

Once we determined the time of spiking of individual neurons, we computed heat maps of the spike rate of each neuron in a fashion similar to previous studies in bats (Yartsev and Ulanovsky, 2013). Briefly, we first binned the spike rate data into spatial locations 10 cm × 10 cm × 10 cm voxels in size. Within sessions, we then employed an adaptive binning procedure to ensure that each bin had an occupancy time of at least 1-s. The 3D bins were then smoothed using a filter 25 cm × 25 cm × 25 cm in size. We then instituted a minimum place field size of a 40 cm × 40 cm × 40 cm on the smallest continuous volume of space eliciting activity at spike rates greater than 50% of the maximum rate. If place field sizes were less than this 3D volume, they were excluded from analysis. 2D place field heat plots were constructed by collapsing along one of the axes (e.g., XY or XZ).

#### Significance Testing of Place Maps

We performed two separate shuffle analyses to test whether hippocampal place fields were significantly localized to a particular area of the room. In the first analysis, we counted the number of spikes generated by each neuron during a session. We then computed a spike train with the same number of overall spikes, but with randomized spike times. With this shuffled spike train, we then recomputed place fields. In the next step, we calculated the size and location of the shuffled place field maps eliciting spikes greater than 50% of the maximum rate, as is described above, and then calculated its percent overlap with respect to the actual place field. This procedure was repeated 1,000 times, and from those 1,000 iterations, we counted the number of times the shuffled place field overlapped with at least 75% of the actual place field. If this occurred less than 50 times out of 1,000 (i.e., less than 5%), then that cell was categorized as having a significantly localized place field. We identified 217 neurons out of 341 total neurons recorded that show statistically reliable place fields. As a second method for computing the significant localization of a hippocampal place field, we shuffled the position data with respect to spike times. The rationale behind this analysis is that when spike times are shuffled, bursting activity of a neuron can be abolished, and thus the analysis can under-estimate the localization of hippocampal activity to one place in the room. In order to preserve any burst of spikes, we instead binned the 3D instantaneous position vector (in 5-s bins), and then randomly shuffled the ordering of the bins with respect to the spike times, and computed a shuffled place map. This procedure was performed 1,000 times, and the average spike rate in each 10 cm × 10 cm × 10 cm bin was computed as was done for the actual spiking data. We then subtracted the average shuffled spike rate in each bin from the corresponding bin in the spatial heat plot computed from the unshuffled (real) data. This shuffling technique was adapted from Geiller et al. (2017). We performed this shuffle correction on the 217 neurons that passed the first shuffle correction method outlined above. We then reinstituted the criteria described above that place fields must be at least 40 cm × 40 cm × 40 cm in size on the 3D heatmaps that had been shuffle corrected by subtracting the shuffled average within each corresponding space bin. This correction resulted in the exclusion of 23 neurons from the set of neurons with reliable place fields, with 194 neurons showing statistically reliable place fields in the final data set.

#### Comparing Place Fields and Chances in Sonar Vocal Rate

To measure place field sizes, we quantified the extent of each place field eliciting spikes at a rate above 50% of the maximum spike rate. We computed place field sizes across several conditions: with and without flight obstacles, during times of low or high vocal rate, as well as independently for both the presence of flight obstacles and changes vocal rate. To examine the effects of flight obstacles on place field sizes, we made measurements of place fields with and without the obstacles, and then performed a Wilcoxon signed-rank test on the paired data to test for significant differences. For the effects of vocal rate on hippocampal neuron activity, we determined whether changes in PI influenced the sizes of place fields. This analysis was performed for data collected in the presence of obstacles for Bat 1 and Bat 2. We first categorized all vocal data into two separate groups: a long PI group, and a short PI group (greater/less than 35 ms). We then clipped out windows of time in the neural activity that corresponded to periods of long PI and periods of short PI. The windows began at the time onset of each vocalization, and extended for 100 ms after the vocalization, but we applied a flexible window size criterion with respect to the PI of sonar vocalizations. For short PI vocalizations (and less frequently for longer PI vocals), it was often the case that another vocalization was produced within the initial 100-ms window, which started at the onset of the identified vocalization. When this occurred, the window was extended to include any subsequent vocalizations until there were no additional vocalizations included in the extended 100-ms window. For instance, if two vocalizations were produced at a PI of 30 ms, the initial window would start at the onset of the first vocalization, but would be extended because of the subsequent vocalization 30 ms later. The window would then start at the onset time of the first vocalization, and then extend until 100 ms after the onset of the second vocalization, resulting in a window size of 130 ms in this example. If there were a third vocalization within 100 ms of the second vocalization, then the window would be extended again until 100 ms after the third vocalization. This process continued until there were no vocalizations within a 100-ms window after the last vocalization in the sequence. Once these windows of neural activity were extracted, we then used the same 1-s occupancy criteria in order to determine the appropriate 3D volume bin size, and place fields were constructed as outlined in the above section. Place field sizes were defined as the region of space greater than 50% of the maximum firing (2D or 3D) as was done previously in hippocampal recordings from the Egyptian fruit bat (Yartsev and Ulanovsky, 2013), and significant differences in place field sizes for short and long PI were tested using a two-tailed *t*-test. A two-tailed *t*-test was used because the data passed the Lilliefors Test for normal distribution.

In order to independently examine the effects of obstacles and sonar vocal rate on place field sizes, we ran paired sessions with Bat 3, one in the presence of flight obstacles and one without obstacles. Because this bat flew in a restricted flight corridor, there was often enough spatial coverage to examine the effects of both short and long PI vocalizations within a session. We could therefore run paired sessions with and without obstacles at the same recording site in the hippocampus to independently examine vocal rate and obstacle influences on place field size. A Wilcoxon signed-rank test was then run on the paired data to determine significant differences.

## Results

Here, we report on hippocampal theta rhythm and place fields of single neurons in animals flying in 3D volumetric space. Data come from hippocampal recordings from three big brown bats (*Eptesicus fuscus*), navigating an experimental test room, with and without obstacles. The animals were released by hand, and most trials ended when the bats landed on a wall, but on rare occasion (<10% of trials), the bats would land on the floor. Bat 1, Bat 2, and Bat 3 performed 11, 11, and 16 flight sessions, respectively. Shown in **Figure 1A** is one experimental session from Bat 1. The bat’s 3D flight trajectories are shown in gray, while locations in space (i.e., times) when the bat vocalized are indicated with blue circles. The flight obstacles are omitted from this figure for clarity. Displayed in **Figure 1B** are vocal and neural data from one flight trial of the experimental session shown in **Figure 1A** (selected flight trajectory indicated with a green line). The top trace shows an oscillogram of the vocalizations produced by the bat while navigating across the room. In this selected trial, the bat altered its vocal repetition rate as it navigated across the room and encountered the flight obstacles (note arrows indicating time of obstacle encounter on top trace, inset displays vocalizations around the time of passing the second flight obstacle). The middle and bottom traces show neural data collected on neighboring channels of a single tetrode during the same trial. The time-expanded portion (red box) of the neural trace shows the output of our two sorting methods (spike amplitude clustering and wavelet-based clustering, see “Materials and Methods”). Different neurons are identified by different colors, with four neurons indicated in the time-expanded neural trace (i.e., red, blue, green, brown asterisks).

As the bats flew within the experimental test room, they encountered flight obstacles, performed aerial maneuvers, and prepared to land. While the bats were performing these behaviors, they made natural adjustments to the rate of sonar vocal production. Shown in **Figure 2A** are trials of Bat 3 flying along a partitioned corridor in the test room (flight paths in blue, corridor shown as light gray shading). Indicated in dark gray are the three flight obstacles hung from the ceiling (left to right, a square pyramid, a cube, and a sphere). These obstacles were hung in order to encourage adaptive vocal behaviors from the bat, and specifically, alterations to the bat’s vocal rate. In **Figure 2B** is a quantification of the Bat 3’s control of vocal rate by analyzing *sonar pulse interval* across all trials, on all days flying with and without obstacles (*n* = 52255 vocalizations). PI is defined as the time delay between the onset of one vocalization to the onset of the next vocalization, with shorter PI’s indicating higher vocal rates (1/PI = vocal rate).

**FIGURE 3:**
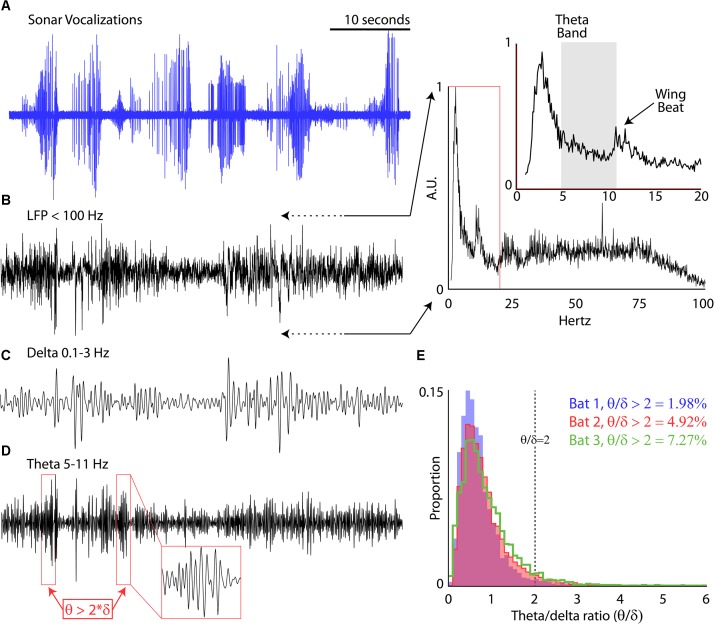
Prevalence of theta rhythm. **(A)** Sonar vocalizations from six consecutive trials of Bat 3 navigating the experimental flight room. **(B)** Local field potential (LFP, <100 Hz) recorded in the hippocampus during the six flights shown in **(A)**. Inset, power relationship across frequencies ranging from 1 to 100 Hz (and 1–20 Hz in expanded portion, *y*-axis shown at same scale). **(C)** Band-pass delta rhythm of the LFP (0.1–3 Hz) during the six flights shown in **(A)**. **(D)** Band-pass theta rhythm of the LFP (5–11 Hz) during the six flights shown in **(A)**. Red boxes identify bouts of increased theta, defined as amplitudes greater than two times the amplitude of the delta band. Inset, zoomed few of one theta bout. **(E)** Proportion 1 s bins where theta bouts occurred for each bat during all experimental sessions (Bat 1, in blue, 11 sessions; Bat 2, in red, 11 sessions; Bat 3, in green, 16 sessions).

While the bats performed the flight tasks, chronic neural recordings were wirelessly transmitted from the hippocampus to a receiver mounted in the center of the room (TBSI). We recorded hippocampal activity using a 4-tetrode array (i.e., 16-channels across tetrodes) mounted on a microdrive. We first examined the prevalence of the theta band (5–11 Hz) in the LFP of our chronic recordings for each session. Shown in **Figure 3A** are vocalizations produced by Bat 2, displayed as oscillograms, across six consecutive flights around the experimental test room. In **Figure 3B** is the simultaneous LFP (<100 Hz) recorded during these six flights. The inset of **Figure 3B** displays the power-frequency relationship of the LFP while the bat was in flight. There is a prominent peak at 11–13 Hz (noted in time expanded figure) indicative of the wing beat of the big brown bat (Norberg and Norberg, 2012). Interestingly, there is no prominent peak in the theta range (5–11 Hz, indicated with a gray box). By band-pass filtering the LFP between 5 and 11 Hz, we specifically examined changes in the theta rhythm over time. Unlike rodents (Buzsáki, 2002), the theta rhythm in the big brown bat does not occur continuously, but instead in bouts. We defined theta bouts by determining when the theta band (5–11 Hz) was two times greater that the delta band (0.1–3 Hz) across 1-s bins. Examples of the delta band and theta band are shown in **Figures 3C,D** that correspond to the data shown in **Figures 3A,B**. Theta bouts are indicated with red boxes in the **Figure 3D**. We found that for each bat the theta rhythm is intermittent, occurring between approximately 2 and 7% of the trial time (**Figure 3E**) for the three bats in our study while they navigated around the flight room.

**FIGURE 4:**
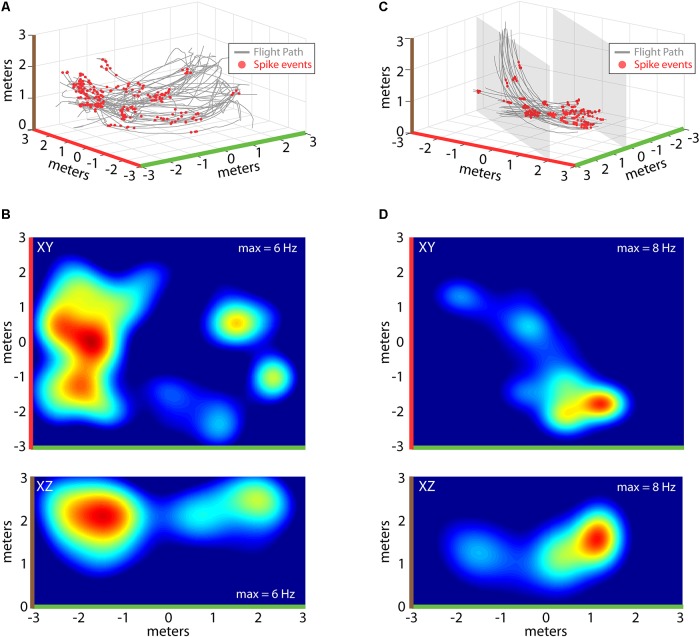
Construction of 2D place fields. **(A)** All flights from one experimental session of Bat 2. Flight paths are shown in gray, locations of spike events from a single hippocampal neuron are shown with red circles. Note colors indicating X, Y, and Z axes (green, red, and brown, respectively). **(B)** Top, heat map of spike rate at different XY locations in the experiment session shown in **(A)**. Maximum spike rate (warmer colors = higher spike rates) is identified at the top right of each panel, green and red highlights on X and Y axes indicate orientation of this panel with respect to **(A)**. Bottom, heat map of spike rate at different XZ locations for the experimental session shown in **(A)**. Colors as in previous panels (note brown color highlighting *z*-axis). **(C)** All flights from one experimental session of Bat 3. For this bat, its flight paths were restricted to a narrow corridor (6 m × 2 m × 3 m) by netting (shown as gray shading). Other colors as in **(A)**. **(D)** Top, heat map of spike rate at different XY locations in the experiment session shown in **(C)**. Bottom, heat map of spike rate at different XZ locations for the experimental session shown in **(C)**.

Recordings of spiking activity were also collected from the three bats, totaling 341 isolated neurons (147 neurons from Bat 1, 107 neurons from Bat 2, and 64 neurons from Bat 3), with 194 neurons showing a localized increase in activity over a restricted region of allocentric space (see “Materials and Methods” for validation steps). Shown in **Figure 4** are example place-fields of neurons recorded from Bat 1 (left) and Bat 3 (right). **Figure 4A** displays flight paths (gray lines) and locations of spike events of a single hippocampal neuron (red circles) for one experimental flight session from Bat 1. Spiking activity of this neuron was highest at one location in the room, near the *X, Y, Z* coordinate: [-2, 0, 2]. To visualize where in the flight room the neuron in **Figure 4A** increased its activity, 2D spike-rate heat plots were constructed. The XY projection of this neuron’s spike-rate heat plot is shown in the top panel of **Figure 4B**. Highlighting colors on the *X*-axis (green) and *Y*-axis (red) indicate the orientation of this heat plot with respect to the 3D representation in **Figure 4A**. In this figure, the spike rate of the neuron has been binned into 25 cm^2^ bins, and then convolved with a 2D Gaussian. Red indicates locations with the highest spike rate (i.e., 6 Hz), whereas blue indicates locations with a low, or zero, spike rate. The bottom panel of **Figure 4B** shows the XZ projection of the same data, indicating where in elevation this neuron fired maximally (brown and green highlighting colors on axes indicate the orientation with respect to **Figure 4A**). Shown in **Figure 4C** are data from a hippocampal neuron recorded in Bat 3 flying within the restricted corridor along the diagonal of the flight room. As in **Figure 4A**, flight trajectories are shown in gray, and the 3D locations of spike events are shown with red circles. For this neuron, there was an increase in spiking activity when the bat was near one end of the corridor (position [1, -2, 1.5] in *X*, *Y*, *Z* coordinates). 2D heat maps for this same neuron are shown in **Figure 4D**. The top panel of **Figure 4D** shows the XY projection of this neuron’s spike rate heat plot, and the bottom panel shows the XZ projection (colors highlighting axes in **Figure 4D** indicate orientation with respect to **Figure 4C**). For both neurons shown in **Figures 4A,B** and **Figures 4C,D**, the highest spike rates occurred when the bat was in a restricted region of space, but there were multiple locations that showed spiking activity at a rate greater than zero.

**FIGURE 5:**
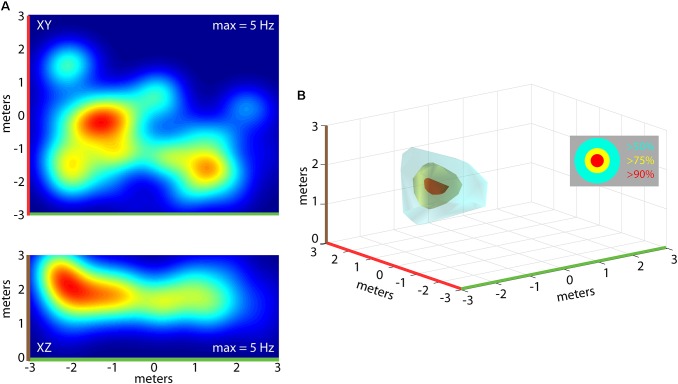
Construction of 3D place fields. **(A)** Top, heat map of spike rate for one neuron during an experimental flight session for Bat 1 (warmer colors = higher spike rates, max rate shown at top right of panel). Shown in the top panel is the XY projection of the neuron’s firing rate. Bottom, the XZ projection for the same neuron shown in the top panel. **(B)** 3D rendering of the place field for the neuron shown in **(A)**. 3D surfaces are plotted identifying the locations in space where the neuron fired at 90% of the maximum rate (red), 75% of the maximum rate (yellow), and 50% of the maximum rate (cyan). Color highlights on axes indicate orientation of 2D heat maps in **(A)**.

Importantly, the bats traversed the 3D volume of the flight room, and we determined whether neurons in hippocampus of the free-flying big brown bat coded for unique 3D positions in allocentric space. For single neurons, we constructed 3D clouds of spike rate activity by binning spike rates in 3D volumes of space (see “Materials and Methods” for validation steps). This is shown for one neuron from Bat 1 in **Figure 5**. Shown in **Figure 5A** are the XY (top) and XZ (bottom) projections of the spike rate heat maps. As in previous figures, note the color-coding of the axes in this panel, and the corresponding color-coding of **Figure 5B**. Displayed in **Figure 5B** is a 3D rendering of the spike rate for the neuron shown in **Figure 5A**. The red cloud indicates the boundaries of space eliciting activity at greater than 90% of the maximum spike rate; yellow indicates spatial locations eliciting spike rates greater than 75% of the maximum spike rate; and cyan colors indicate spiking activity greater than 50% of the maximum firing rate. For this neuron, there was a restricted region of space, centered at [2, 0, 2] meters, where the firing was maximal; additionally, a volume of space the size of 3.20 cubic meters around this coordinate showed activity at a spike rate greater than 50% of the maximum rate.

**FIGURE 6:**
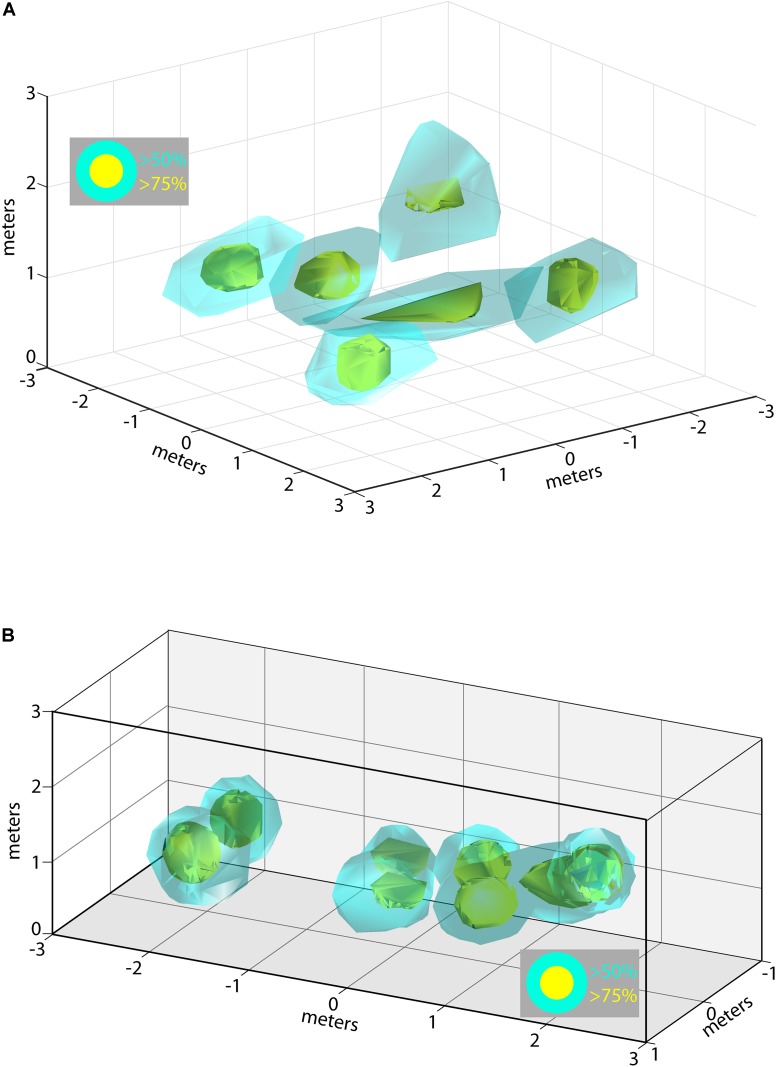
Composite of 3D place fields in one recording session. **(A)** Locations of 3D place fields for six different neurons recorded during one experimental session for Bat 2. Yellow clouds indicate the boundaries of spatial locations eliciting firing rates greater than 75% of the maximum firing rate; cyan indicates locations eliciting firing rates greater than 50% of the maximum rate. **(B)** Locations of 3D place fields for eight different neurons recorded during one experimental session for Bat 3. Note that the flight paths for Bat 3 were restricted to a narrow corridor. Colors as in **(A)**.

During some experimental sessions we were able to record from multiple neurons simultaneously, allowing us to examine how the 3D allocentric space of the flight room is represented across a pool of neurons in the hippocampus. Shown in **Figure 6A** are the locations and 3D spike rate clouds of six different neurons recorded from Bat 2 during flight session 7. Displayed are the locations in 3D space driving the neuron at greater than 50% (cyan), and greater than 75% (yellow), of the maximum spike rate. These six neurons’ 3D place clouds span most of the volume of space in which the bat flew. Similar data is shown for Bat 3 in **Figure 6B** from experimental session 3. In this figure, the spike rate clouds for eight neurons (colors as in **Figure 6A**) are shown within the edges of the restricted flight corridor. We again find that 3D place fields seem to be uniformly distributed throughout the volume of the bat’s flight space.

**FIGURE 7:**
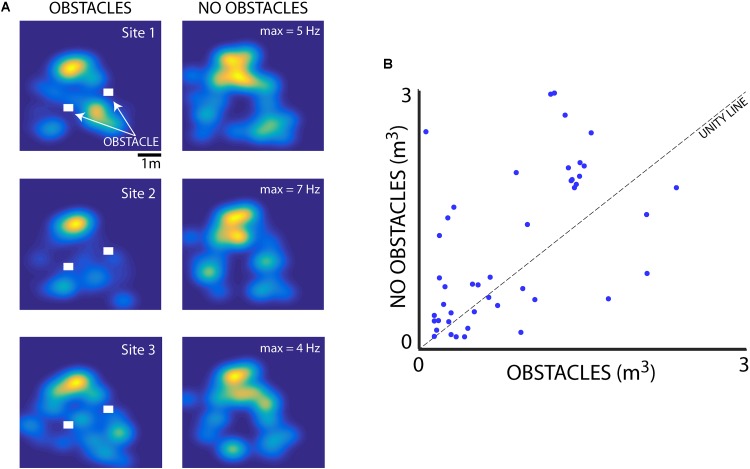
Effect of flight obstacles on place field size. **(A)** Left column, XY heat maps of firing rates for three different neurons recorded in Bat 2 in the presence of 3D flight obstacles in the experimental test room. Obstacles are shown as white rectangles. Right column, the activity of the same three neurons as shown in left column, but when the obstacles were removed from the room. Maximum firing rates for each neuron are shown on the right column heat maps. **(B)** Comparison of place field size (m^3^) for neurons in the presence and absence of flight obstacles for Bat 1 and Bat 2. Place fields are significantly smaller in the presence of obstacles than when obstacles are absent (*n* = 49, *p* = 0.019, rank-sum test).

We also examined the influence of flight obstacles and the bats’ vocal rate on the sizes of place fields within the experimental test room. We first determined whether the presence of flight obstacles affected hippocampal place field tuning. Shown in **Figure 7** are hippocampal data collected from neurons in the presence and absence of flight obstacles. To collect these data, paired sessions were run with Bats 1 and 2, one with two flight obstacles near the center of the room (indicated by white rectangles), and then another session was run at the same recording location with the flight obstacles removed (the session order was randomized). Shown in **Figure 7A** are data from three neurons in Bat 2 that were recorded during paired sessions with and without the flight obstacles. These three example neurons show that hippocampal place fields were smaller when obstacles were present than when obstacles were absent. Additionally, there were some changes in the place field locations when the flight obstacles were added/removed. For instance, the hippocampal neuron shown in the top panel of **Figure 7A** fired near the right-most flight obstacle when it was present, but the neuron showed no activity at this location when the obstacle was removed. Presented in **Figure 7B** are pairwise data from 49 neurons collected with and without obstacles from recording sessions with Bat 1 and Bat 2. These summary data show that the size of place fields (as defined as the area greater than 50% of the maximum firing rate) was significantly smaller when the bat was flying in the presence of obstacles (*p* = 0.019, signed-rank test).

**FIGURE 8:**
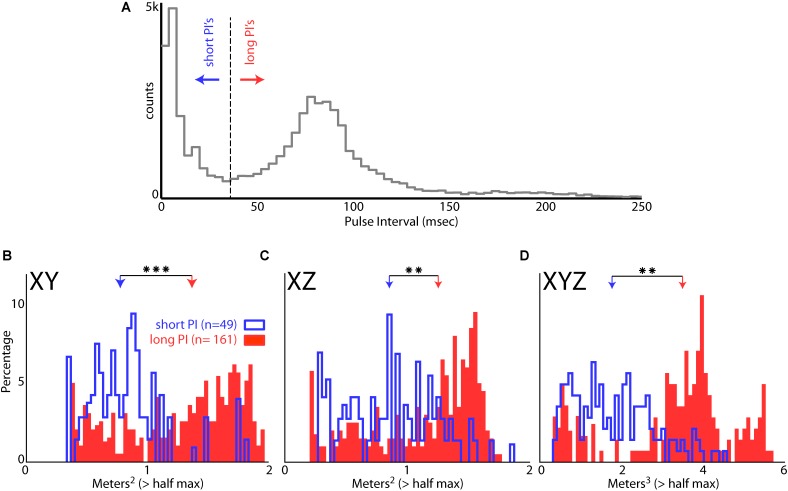
Effect of vocal rate on place field size. **(A)** Distribution of pulse intervals (PIs) for Bat 1 and Bat 2. Note the bimodality of the distribution, with the minima between modes falling at a PI of 35 ms. **(B)** Size of place fields in the XY dimension (defined as m^2^ greater than the half of the maximum firing rate) when the bats were emitting vocalizations at a short PI (blue, less than 35 ms PI’s, *n* = 49 neurons), and when the bats were emitting vocalizations at a longer PI (red, greater than 35 ms PI’s, *n* = 161 neurons). There is a significant decrease in place field size when the bats are emitting vocalizations at a short PI (*p* = 6.54e-12, two-tailed *t*-test; arrows indicate mean values). Right, comparison of mean place field size when the bat was producing vocalizations at a short PI (left, blue) and long PI (right, red) vocalizations. **(C)** Size of place fields in the XZ dimension when the bats were emitting vocalizations at a short PI (blue), and long PI’s (red). There is a significant decrease in place field size when the bats are emitting vocalizations at a short PI (*p* = 3.54e-6, two-tailed *t*-test). **(D)** Size of place fields in the XYZ dimensions when the bats were emitting vocalizations at a short PI (blue), and long PI’s (red). There is a significant decrease in place field size when the bats are emitting vocalizations at a short PI (*p* = 1.72e-8, two-tailed *t*-test). ^∗∗^ indicates *p* < 1e-5, ^∗∗∗^ indicates *p* < 1e-10.

Bat 1 and Bat 2 flew in the presence of flight obstacles in the entire volume of the large test room. We analyzed data from these two bats in the presence of flight obstacles to control for possible effects of adding/removing the obstacles on place field sizes. Hippocampal data from within an experimental recording session were then pooled into two groups: time periods when the bats were vocalizing at a high rate (short PI), and time periods at a lower rate (long PI) (see “Materials and Methods”). **Figure 8A** shows the distribution of PI’s for all sessions of Bat 1 and Bat 2 when they flew in the presence of flight obstacles. This distribution is bimodal, with a local minimum centered at a PI of 35 ms, which then served as the cut off between short and long PI’s. **Figure 8B** displays the sizes of hippocampal place fields in the XY projection of the room when the bats produced vocalizations at a short PI (blue), and when the bats produced vocalizations at a longer PI (red). The sizes of place fields (as defined by the area of space eliciting spikes greater than 50% of the max firing rate) were significantly smaller in the XY, XZ, and XYZ dimensions when the bats produced vocalizations at a short PI, i.e., increased vocal rate (**Figure 8B**, XY dimension,*p* = 6.54e-12; **Figure 8C**, XZ dimension, *p* = 3.54e-6, XYZ dimension, *p* = 1.72e-8, two-tailed *t*-test). These results suggest that place field sizes are reduced when bats produce vocalizations at a higher rate (shorter PI’s).

**FIGURE 9:**
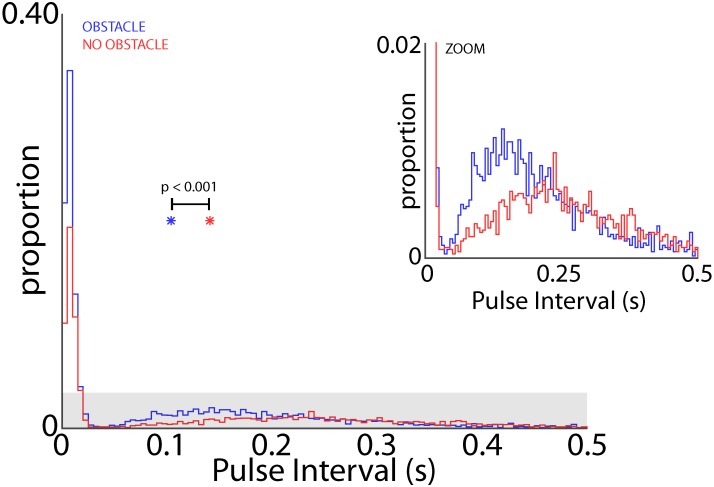
Effect of obstacles on vocal rate. Distribution of pulse intervals (PIs) for all bats in the presence of flight obstacles (blue) and in the absence of flight obstacles (red). Inset shows gray shaded region in larger panel. PI is significant smaller when bats flew in the presence of flight obstacles (*p* < 0.001, permutation test). Colored asterisks indicate the mean PI’s for the obstacle (blue) and no obstacle (red) conditions.

When the bats flew in the experimental test room, they adaptively modified their sonar vocal rate with respect to objects in the environment (i.e., adaptive changes in vocal PI as shown in **Figure 2**). As such, we examined whether the obstacles themselves changed the dynamics of adaptive vocal control. Shown in **Figure 9** are data from all bats when they flew in the presence of flight obstacles (blue) and when the obstacles were removed (red) across all experimental sessions. These data show that vocal PI is significantly reduced when the bats flew in the presence of flight obstacles (permutation test, *p* < 0.001), indicating that bats increased sonar vocal rate (the inverse of PI) when flying in a more cluttered environment. Considering that place field sizes were smaller in the presence of flight obstacles (**Figure 8**), and that flight obstacles also had an effect on vocal rate, we then examined the independent effects of vocal rate and flight obstacles on place field size.

**FIGURE 10:**
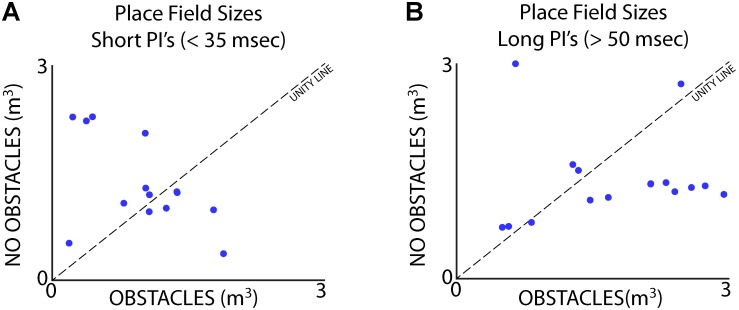
Separation of vocal rate and flight obstacle effects on place field size. **(A)** Size of place fields with and without flight obstacles when Bat 3 was producing vocalizations at a short PI (less than 35 ms). There is no consistent significant difference in place field size across the two conditions (signed-rank, *n* = 19 neurons, *p* > 0.05). **(B)** Size of place fields with and without flight obstacles when Bat 3 was producing vocalizations at a long PI (greater than 50 ms). There is also no consistent significant difference in place field size across the two conditions (signed-rank, *n* = 16 neurons, *p* > 0.05).

Data from Bat 3, which flew in a restricted corridor, allowed us to independently analyze the effects of vocal rate and the presence of obstacles for a small subset of neurons. We ran three paired sessions – one session in the presence flight obstacles (**Figure 2A**), and one session without the flight obstacles – while recording from the same site in the hippocampus across the paired sessions. We then sorted each session by vocal PI, with the short PI group including all PI’s less than 35 ms, and the longer PI group including all PI’s greater than 50 ms. The sizes of place fields were compared across the presence and absence of flight obstacles for times of short PI vocalizations (**Figure 10A**), as well as for periods of long PI vocalizations (**Figure 10B**). We found that when controlling for sonar vocal rate, there was no consistent and significant effect of the presence flight obstacles on place field size. This is true when controlling for both short and long PI vocalizations (**Figures 10A,B**, signed-rank tests, *p* > 0.05 in both cases, *n* = 16).

## Discussion

The hippocampus has been shown to play a role in a variety of memory-guided tasks, including operant conditioning (Ishikawa et al., 2014), and other associative learning tasks (Mattfeld and Stark, 2015; Oh and Disterhoft, 2015; Epsztein and Koenig, 2017; Liu et al., 2017). A great deal of laboratory studies have used rodent models to investigate the role of the hippocampus in memory-guided navigation (Moser et al., 2017). Comparative work in non-human primates (Stewart and Fox, 1991; O’Mara et al., 1994; Rolls, 1999) and echolocating bats have added to our knowledge of hippocampal function, identifying common mechanisms across animals, as well as noteworthy differences. Data from diverse animal systems provides a broad perspective on hippocampal function, and our recordings from the flying big brown bat adds to this literature by demonstrating the influence of active sensing on hippocampal place field tuning.

Many models of hippocampal function rely on theta oscillations (Robinson, 1980; Bland and Oddie, 2001; Hasselmo, 2005). In the rodent, the theta rhythm is robust and continuous, with increases in theta power tied to exploratory behaviors in the environment (Vertes, 2005). Recordings from the primate hippocampus have demonstrated intermittent bouts of theta (Stewart and Fox, 1991), and the same is reported for freely moving humans (Aghajan et al., 2017). Work in the hippocampus of crawling big brown bats (Ulanovsky and Moss, 2007), and flying Egyptian fruit bats (Yartsev and Ulanovsky, 2013) also report bouts of theta, and here we analyzed LFP in flying big brown bats, whose wingbeat rate is about 12 Hz, outside the theta band (Norberg and Norberg, 2012). Our data corroborates prior reports in the Egyptian fruit bat and primate, showing only intermittent bouts of theta while the bat explored its environment in flight. These data motivate future studies in other species to determine the generalized role of theta in hippocampal function.

We mapped hippocampal place cells in the free-flying big brown bat and found that neurons are tuned to 3D locations in space, in agreement with prior studies of the Egyptian fruit bat (Yartsev and Ulanovsky, 2013). While big brown bats and Egyptian fruit bats are both in the order Chiroptera, they belong to different suborders and rely on laryngeal and lingual echolocation, respectively (Lei and Dong, 2016). Collectively, data from free-flying bats highlight the importance of an animal’s natural navigation behaviors to the features of place cell tuning, particularly with respect to its 3D environment and the adaptive behaviors employed during navigation. Prior work in rodents has hinted at 3D allocentric place coding in the hippocampus (McNaughton et al., 2000; Knierim and McNaughton, 2001), but Jeffery et al. (2013) have argued that 3D place coding arises from a stacking of 2D place cells. We posit that the differences between rodents and bats can be attributed to species-specific navigation behaviors: rodents travel along surfaces, while bats travel through 3D volumetric space (explained in Moss, 2013).

As the laryngeal echolocating bat flies in 3D space and navigates around obstacles, it adapts its sonar call rate (Petrites et al., 2009; Moss and Surlykke, 2010; Falk et al., 2014; Kothari et al., 2014). These changes in the bat’s behavior can be used to investigate the influence of natural sensory sampling on hippocampal spatial representations. Past research on the hippocampus has shown that an increase in sensory information leads to a decrease in hippocampal place field size (Battaglia et al., 2004). Since echolocating bats increase sonar call rate to inspect objects (Petrites et al., 2009; Moss and Surlykke, 2010; Kothari et al., 2014), we could take advantage of this behavior to investigate whether sensory sampling rate modulates place field tuning in these animals. First, we confirmed that the presence of flight obstacles evoked higher call rates in free-flying bats (**Figures 2**, **9**), and then showed that higher call rates resulted in a decrease in hippocampal place field size (**Figure 8**). From these data, we conclude that higher sensory sampling rates lead to sharper place fields in the hippocampus.

Models of hippocampal spatial memory formation posit that egocentric sensory information guides allocentric representations through the locations of borders (O’Keefe and Burgess, 1996; Hartley et al., 2000) or landmarks (Strösslin et al., 2005; Sheynikhovich et al., 2009), with both models predicting that higher sensory acuity during place field formation will lead to smaller place fields. In support of this model, small changes in an animal’s sensory environment have been shown to lead to a variety of changes – from modest to robust – in hippocampal place field dynamics, providing further evidence that sensory information can affect spatial representation in the hippocampus (reviewed by Knierim, 2015). Our results on the relationship between sensory acquisition rate and place field sizes lend evidence to these sensory-based models of spatial representation.

Considering the importance of sensory sampling to establish the allocentric layout of an environment (O’Keefe and Burgess, 1996; Sheynikhovich et al., 2009; Geva-Sagiv et al., 2015), studies have experimentally examined the relationship between the availability of sensory information and the dynamics of hippocampal place fields. In one study, the availability of sensory cues (visual, auditory, olfactory, and tactile) was altered to varying degrees while assaying the influences on hippocampal place coding (Battaglia et al., 2004). Place fields were compared under sensory cue-rich and sensory cue-sparse conditions, and the authors found that place field sizes were significantly smaller when more cues were available. In a more recent study, experimenters altered the availability of visual landmarks in rats by running sessions in the light and in the dark, and showed that when visual stimuli were available to the rat, place fields were smaller (Zhang et al., 2014). These studies manipulated the availability of sensory cues to the animal, but these studies did not control for sensory sampling effects on place field size. This is a straight-forward analysis in the echolocating bat because the animal’s vocal rate provides a direct measure of the flow of sensory signals available to the animal. By utilizing the echolocating bat as a model to investigate the effects of sonar call rate on place field size, we provide preliminary data showing a direct link between sensory sampling, and the sharpness of hippocampal spatial representation.

In more recent work, temporary increases in the bat’s sonar vocal rate are shown to correlate with time periods of increased spatial attention (Petrites et al., 2009; Falk et al., 2014; Kothari et al., 2018). Past work in rodents has also investigated dynamic changes in hippocampal place cell activity with respect to changes in the animal’s attentional state (Fenton et al., 2010). In Fenton et al.’s (2010) study, rats were trained to either forage for food, or find a goal location, with the hypothesis that searching for a goal location would invoke a heightened attentional state. Hippocampal activity was then analyzed for overdispersion, or an increase in the variability of hippocampal place cell activity over what would be predicted by mean firing rate alone (Fenton and Muller, 1998; Jackson and Redish, 2007). The authors found that when the animal was navigating to a goal location instead of randomly foraging, the amount of overdispersion significantly decreased, resulting in sharper and more reliable place fields (Fenton et al., 2010). The authors hypothesize that the navigation task invokes an increase in spatial attention compared to the random foraging task, and this change in attentional state decreased overdispersion in place cell tuning. These results are related to those we report here: when the bats increased their sonar vocal rate to more closely inspect the environment, place fields became less diffuse and more localized in space. Taken together, these studies identify possible effects of spatial attention on hippocampal place cell activity.

## Conclusion

Our knowledge of hippocampal function has benefited greatly from comparative work in rodents, birds, primates, and bats. Here, we have reviewed past studies of bat hippocampal representation and pointed to gaps in the literature. As a first step toward bridging these gaps, our data show only bouts of hippocampal theta rhythm in free-flying big brown bats. In addition, we present new data showing changes in 3D hippocampal place field tuning with the rate of sensory sampling through echolocation. Our experiments uncovered hippocampal dynamics, which may also operate in animals that sense the environment through other modalities. Further comparative research can serve to elaborate on species-specific specializations and general principles of hippocampal representation.

## Author Contributions

MW designed and conducted the experiments, performed the analysis, and wrote the manuscript. CY helped to conduct the experiment and write the manuscript. CM designed the experiment, provided input for the analysis, and helped to write the manuscript.

## Conflict of Interest Statement

The authors declare that the research was conducted in the absence of any commercial or financial relationships that could be construed as a potential conflict of interest.

## Footnotes

1 https://www.homedepot.com/p/7-ft-x-100-ft-Deer-Block-Netting-889553A/202267888
